# Artificial Intelligence-Based Automated Assessment of the Four-Chamber View in Fetal Cardiac Ultrasound Videos

**DOI:** 10.3390/bioengineering13030303

**Published:** 2026-03-05

**Authors:** Naoki Teraya, Masaaki Komatsu, Katsuji Takeda, Kanto Shozu, Naoaki Harada, Reina Komatsu, Akira Sakai, Rina Aoyama, Mayumi Kaneko, Ken Asada, Syuzo Kaneko, Kazuki Iwamoto, Akitoshi Nakashima, Ryu Matsuoka, Akihiko Sekizawa, Ryuji Hamamoto

**Affiliations:** 1 Department of Obstetrics and Gynecology, Showa Medical University School of Medicine, 1-5-8 Hatanodai, Shinagawa-ku, Tokyo 142-8666, Japan; 2 AI Medical Engineering Team, RIKEN Center for Advanced Intelligence Project, 1-4-1 Nihonbashi, Chuo-ku, Tokyo 103-0027, Japan; 3 Division of Medical AI Research and Development, National Cancer Center Research Institute, 5-1-1 Tsukiji, Chuo-ku, Tokyo 104-0045, Japan; 4 Department of Obstetrics and Gynecology, University of Toyama, 2630 Sugitani, Toyama 930-0194, Japan; 5 Department of NCC Cancer Science, Biomedical Science and Engineering Track, Graduate School of Medical and Dental Sciences, Institute of Science Tokyo, 1-5-45 Yushima, Bunkyo-ku, Tokyo 113-8510, Japan; 6 Digital Health Platform Development Office, Healthcare Business Unit, Fujitsu Japan Ltd., 1-5 Omiya-cho, Saiwai-ku, Kawasaki 212-0014, Japan; 7 Artificial Intelligence Laboratory, Fujitsu Ltd., 4-1-1 Kamikodanaka, Nakahara-ku, Kawasaki 211-8588, Japan

**Keywords:** artificial intelligence, fetal cardiac ultrasound screening, four-chamber view, biometric parameters, clinical comparison study

## Abstract

The clinical application of artificial intelligence (AI) can provide technical support for examiners and improve obstetric workflow efficiency. In this study, we developed AI models that automatically extract the four-chamber view (4CV) from fetal cardiac ultrasound videos and compute the cardiothoracic area ratio, cardiac axis, and cardiac position for prenatal screening of congenital heart disease. Fetal cardiac ultrasound videos from 301 patients in the second trimester were analyzed. The 4CV was automatically extracted using YOLOv7, followed by image segmentation with UNet 3+ and SegFormer, after which automated parameter calculation and estimation were performed. A clinical comparison study involving 22 obstetricians was conducted to evaluate the screening performance of the AI models. The models demonstrated stable performance in both normal and abnormal cases, including examinations acquired using different ultrasound systems. Furthermore, the AI models achieved screening performance comparable to that of expert obstetricians. These findings indicate that the proposed AI framework enables reliable 4CV extraction and accurate biometric parameter computation. This fully automated approach has the potential to reduce missed abnormalities and improve the consistency of fetal cardiac ultrasound screening.

## 1. Introduction

Ultrasound imaging is widely used, particularly in obstetrics, because its noninvasive and real-time capabilities impose minimal burden on both the mother and fetus [1,2]. However, ultrasound image quality depends heavily on the examiner’s expertise because of the manual nature of image acquisition and the presence of acoustic shadows [3]. Artificial intelligence (AI) technologies for medical image analysis have advanced rapidly in recent years [4,5,6]. To mitigate the limited availability of medical image data, approaches such as ensemble learning, transfer learning, and large multimodal models have been widely adopted [7,8,9,10]. In addition, AI is expected to help address workforce shortages and standardize the accuracy of ultrasound examinations [11,12,13]. Previous studies have shown that, after a sonographer manually selects an appropriate four-chamber view (4CV), a representative transverse cardiac plane, AI systems can measure the biparietal diameter, abdominal circumference, and femur length to estimate fetal body weight [14]. AI-based methods have also been used to evaluate the fetal central nervous system by measuring the lateral ventricles and cerebellar width in fetal brain cross-sections to identify abnormalities [15,16]. In adults, semiautomated methods for cardiovascular echocardiography are revolutionizing the field of cardiology. AI has been clinically applied to estimate left ventricular ejection fraction by recognizing the left ventricle in the 4CV [17]. A prospective trial with pre- and post-sequential allocations reported that AI-assisted focused cardiac ultrasound led to a higher rate of treatment plan changes [18]. A deep learning method was proposed for the fully automated quantification of the calcific burden in high-resolution intravascular ultrasound images [19].

Fetal cardiac ultrasound screening is useful to detect congenital heart disease (CHD), a condition that affects approximately 0.8–1.2% of all births and often requires advanced postnatal medical care. Although prenatal detection has improved with advances in ultrasound technology, the global prenatal diagnostic rate of approximately 60% remains insufficient [20,21,22,23]. Because of the shortage of specialists in fetal cardiology, substantial variability in diagnostic performance exists among examiners. Training clinicians to perform detailed cardiovascular examinations requires considerable time, in part because opportunities for screening are limited and because the fetal heart is small and often poorly visualized due to acoustic shadows or fetal motion. During fetal cardiac ultrasound screening, examiners typically observe several standard transverse cardiac planes, including the 4CV, three-vessel view (3VV), and three-vessel tracheal view [24]. A retrospective quality assessment of fetal cardiac ultrasound images in 92 patients with severe CHD reported significant differences in image quality between patients with prenatally detected and undetected CHD [25]. In addition, the cardiothoracic area ratio (CTAR), cardiac axis, and cardiac position (point P) are broadly applicable screening parameters. However, the accuracy of manual measurements of these indices depends on the experience and skill levels of the examiners [26]. When a pregnant woman is examined at a clinic where the examiner can assess only estimated fetal weight, she may not have the opportunity to undergo a comprehensive fetal cardiac evaluation [27]. Therefore, developing AI-based technologies to support screening and to identify pregnant women who require further detailed examination is clinically important.

Few automated methods can extract appropriate frames from ultrasound videos and compute biometric parameters; however, the lack of interpretability of AI-based diagnoses remains a major concern [11]. To assist examiners in fetal cardiac ultrasound screening, we previously proposed a method that visualizes fetal cardiac structures over time using a barcode representation generated by AI [28]. In this study, we aimed to develop an automated AI model that extracts the 4CV from fetal cardiac ultrasound videos acquired during routine prenatal checkups and computes the CTAR, cardiac axis, and point P. To mirror real-world clinical measurements, we employed segmentation models, including UNet 3+ [29] and SegFormer [30]. UNet 3+ is an advanced convolutional neural network (CNN) architecture that has been applied to medical image segmentation. SegFormer is a transformer-based model developed specifically for semantic segmentation. Through a clinical comparison study, we evaluated the performance of these AI models against that of obstetricians in estimating biometric parameters.

## 2. Materials and Methods

### 2.1. 4CV Extraction from Fetal Cardiac Ultrasound Videos

Two processing pipelines were developed: one for extracting 4CV images from fetal cardiac ultrasound videos and another for image analysis. The automatic extraction model developed in our laboratory is based on YOLOv2 for cardiac region detection and was subsequently retrained using YOLOv7 [31]. In this study, YOLOv7 was selected for 4CV extraction because it offers high speed, high accuracy, and continuous updates, which are essential for clinical application. The method for recognizing 18 anatomical sites in the thoracic cavity remained unchanged from the previous model: the crux of the heart, ventricular septum, right atrium, tricuspid valve, right ventricle, left atrium, mitral valve, left ventricle, pulmonary artery, ascending aorta, superior vena cava, descending aorta, gastric vesicle, spine, umbilical vein, inferior vena cava, pulmonary vein, and ductus arteriosus. The extraction model was configured to convert each video into still image sets with a resolution of 480 × 640 pixels at 30 frames per second (fps). The foundation model for target detection is integrated into an AI-equipped software that was approved as a medical device by the Pharmaceuticals and Medical Devices Agency of Japan in July 2024 (approval number: 30600BZX00155000). Based on our previous experimental results, the detection threshold for YOLOv7 was adjusted to enable accurate 4CV extraction suitable for fetal cardiac ultrasound screening. For 4CV recognition, the crux of the heart, right atrium, right ventricle, left atrium, left ventricle, and descending aorta were detected simultaneously, with confidence thresholds set to 0.50, 0.30, 0.30, 0.60, 0.30, and 0.40, respectively. All images meeting these thresholds were extracted from each video, and two images per video were randomly selected for the automated analyses described below.

### 2.2. Dataset

The dataset comprised 301 pregnant women with singleton pregnancies at 18–28 weeks of gestation who underwent fetal ultrasound examinations between 2016 and 2023 at Showa Medical University Hospitals (Supplementary Table S1). Experts or obstetricians with at least 3 years of experience under appropriate supervision performed examinations. Ultrasound examinations were conducted using Voluson^®^ E8, E10, or Expert 22 (GE Healthcare, Zipf, Austria) equipped with 2–6 MHz abdominal transducers, in accordance with established guidelines [32]. Among these, 253 videos from 231 patients with normal fetal cardiac findings acquired using Voluson^®^ E8 or E10 were used to train the models. These sweep videos continuously captured the fetal gastric vesicle and aortic arch over 10–15 s. Each video was divided into frames at 30 fps, and an obstetrician selected one to three images per video that clearly demonstrated an appropriate 4CV, yielding a total of 488 images for training the segmentation models. This dataset was distinct from the dataset used for automated and random 4CV extraction with the preconstructed YOLOv7 model described in Section 2.1.

### 2.3. Model Structure

**Figure 1:**
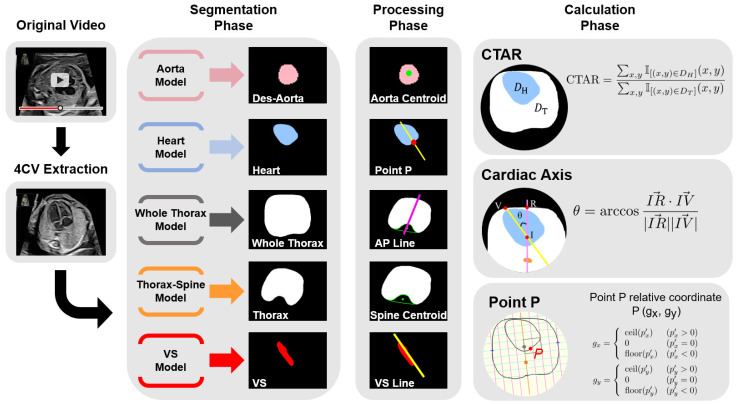
Model overview. The figure shows the workflow from inputting fetal cardiac ultrasound videos to parameter calculation. YOLOv7 detects four chambers of the heart, crux, and descending aorta in the 4CV and randomly extracts set number of images from the image sets over the confidence threshold. Then, the five structures are segmented using the artificial intelligence model. The spine approximated the centroid filled-in hollow of the thoracic segmentation result using a convex envelope. Define a purple line passing through the centroid of the spine and bisecting the area of the whole thorax, and a yellow line passing through the centroid of the ventricular septum in the long axis direction. Finally, programmed calculation and parameter estimation are performed. 4CV, four-chamber view; Des-Aorta, descending aorta; VS, ventricular septum; CTAR, cardiothoracic area ratio.

Figure 1 provides an overview of the proposed model. Segmentation labels for the heart, ventricular septum, whole thorax, thorax, and descending aorta in the 4CV were assigned by an obstetrician. For the spine, the convex-envelope method was used to approximate its centroid by filling the hollow region within the thoracic segmentation where the spine was located. The spine position was therefore represented by its centroid. Subsequently, the following measurements were described, and parameters were calculated using segmentation labels: a line passing through the centroid of the spine and bisecting the area of the whole thorax, line passing through the centroid of the ventricular septum in the long axis direction, and point P according to the program. Point P is defined as the intersection of the line passing through the ventricular septum and the circumference of the heart. Data augmentation was performed due to the limited amount of training data. Next, we segmented the above target area using UNet 3+ and SegFormer and evaluated the performance of the models using mean values of the dice coefficient (mDice). We compared the predictions and labels pixel-by-pixel to determine true positives (TP), false positives (FP), and false negatives (FN); the Dice results are as follows:

(1) $$Dice=\;\frac{TP}{TP+\frac{1}{2}\left(FP+FN\right)}.$$

Based on the predictions of the CTAR, cardiac axis, and point P, the performance of the AI models was evaluated.

### 2.4. Segmentation

All experiments were performed on NVIDIA A100 GPUs using the PyTorch (v2.10.1) framework. Although previous studies have reported results using other CNNs, improvements in speed, accuracy, and response time remain critical for clinical applications. The primary objective of this study was to compare CNN- and Transformer-based segmentation models; therefore, UNet 3+ and SegFormer were selected because they provide high accuracy and inference speed while maintaining low computational cost. Both UNet 3+ and SegFormer are suitable for fetal cardiac ultrasound screening, as reported previously [33]. The feature extraction capacity and computational characteristics of UNet 3+ and SegFormer were as follows: number of parameters, 47.22 and 26.97 MB; floating-point operations (FLOPs), 198.45 and 18.26 GB; inference speed, 96.37 and 91.45 fps; and input image size, 256 × 256 pixels. Both models were trained using segmentation labels for the heart, ventricular septum, whole thorax, thorax, and descending aorta in the 4CV. The following training settings were applied to both architectures: a batch size of 16 and 200 training epochs. The RAdam optimizer was used with a learning rate of 0.0003 and a decay rate of 0.9, and parameters were updated using a momentum-based method. Early stopping was applied if no improvement was observed for 20 consecutive epochs. Data augmentation included horizontal and vertical flipping, random translation, scaling, and rotation, Gaussian blurring, random occlusion of regions up to 75 × 75 pixels, random brightness and contrast adjustments, and the addition of a single random shadow. A hybrid loss function was used, defined as:

(2) $$L_{hybrid}=L_{Focal}+L_{MS-SSIM}+L_{IoU}.$$

*Focal* represents the focal loss [34], *MS-SSIM* represents the multiscale structural similarity index loss [35], *IoU* represents the intersection over union loss. This loss function is used in the base model of UNet 3+, and we adopted it without modification. The same loss function was applied to SegFormer to standardize the training conditions across both models. Receiver operating characteristic (ROC) analyses were performed using Python (v3.10.12).

### 2.5. Parameter Calculations

#### 2.5.1. CTAR

In clinical practice, the heart and whole thorax are commonly approximated as ellipses to calculate the CTAR. In this study, however, contour-based labels were used to enable automatic measurement by the AI model. The numbers of segmented pixels within the thoracic area and heart were counted, and CTAR was calculated as follows:

(3) $$CTAR\;=\;\frac{\sum_{x,y}I_{\left[\left(x,y\right)\in D_{H}\right]}\left(x,y\right)}{\sum_{x,y}I_{\left[\left(x,y\right)\in D_{T}\right]}\left(x,y\right)}.$$

#### 2.5.2. Cardiac Axis

**Figure 2:**
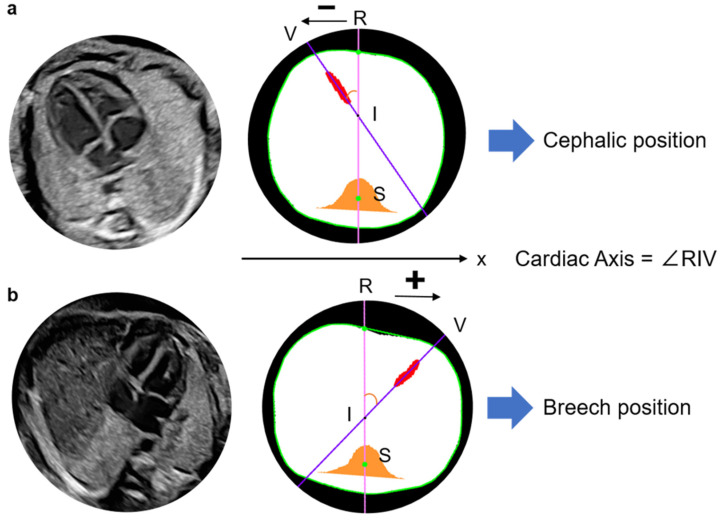
Definition of cardiac axis and recognition method of the fetal cardiac apex. Define the line RS as the *y*-axis and *x*-coordinates of points V and R, which are used to presume the cephalic or breech position in the image. Point V is located at a negative position on the *x*-axis relative to point R and is thus determined to be a cephalic position (**a**). Point V is located at a positive position on the *x*-axis relative to point R and is thus determined to be a breech position. The cardiac axis is calculated as ∠RIV in any fetal position (**b**). R, Intersection of the thoracic circumference with a pink line passing through the spine and bisecting the whole thorax; S, Spine; V, Intersection of a purple line through the centroid of the ventricular septum and the thoracic circumference, closer to the ventricular septum as the apex substitute; I, Intersection of a line through the centroid of the ventricular septum and line RS.

To calculate the cardiac axis, the centroid of the approximated spine was defined as point S (Figure 2). Point R was defined as the point farthest from point S among the intersections between the thoracic outer contour and the line passing through point S that bisects the thoracic area. A line was fitted to the segmented ventricular septum using the least-squares method, and the angle at the intersection point I between this line and line RS was calculated. For the bisecting line RS of the whole thorax, point V was defined as the intersection between the line passing through the centroid of the ventricular septum and the outer contour of the whole thorax on the side closer to the ventricular septum. The model was designed to measure the angle between lines RS and IV regardless of whether the cardiac apex was oriented to the left or right. The angle ∠RIV was defined as the cardiac axis and was calculated as follows:

(4) $$∠RIV\;=\;arccos\frac{\vec{IR}\cdot \vec{IV}}{\left|\vec{IR}\right|\left|\vec{IV}\right|}.$$

**Figure 3:**
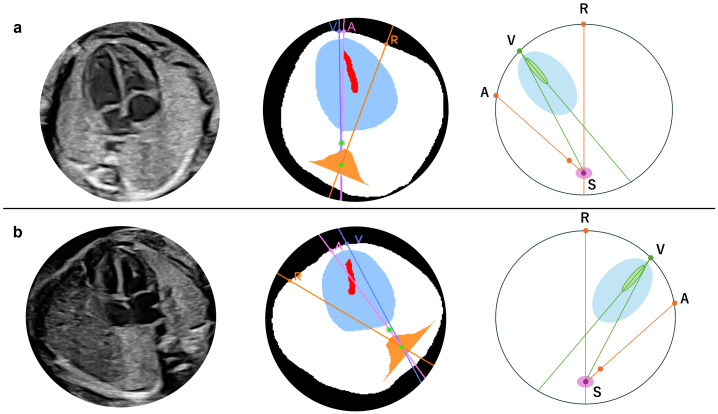
Definition of the apex location and fetal position by the angle in normal case. Cephalic (**a**) and breech (**b**) positions. In either position, the fetal heart of the normal case is described with the degree, drawing a straight line from point S to the other three points, respectively, such as ∠VSA∠RSV + ∠RSA. A, The intersection of the extension pink line connecting the spine and descending aorta with the thoracic circumference as the descending aorta substitution; R, Intersection of the thoracic circumference with an orange line passing through the spine and bisecting the whole thorax; S, Spine; V, Intersection of a line through the centroid of the ventricular septum and the thoracic circumference, closer to the ventricular septum as the apex substitute. The extension purple line passes through S and V.

The fetal cardiac apex direction varies depending on fetal presentation, which is defined as breech when the fetal head is oriented toward the maternal head or cephalic when it is oriented toward the caudal side of the uterus. By examining the x-coordinates of points R and V, the model determines the apex orientation. In a normal fetus in the cephalic position, the apex is located in the left thoracic cavity in the ultrasound view. In the present model, if point V lies in the negative direction relative to line RS, the cardiac apex is classified as being in the left thoracic cavity and is defined as the cephalic position (Figure 3). In cases of CHD in which the apex is located in the right thoracic cavity, the model classifies the orientation as breech. The model, therefore, identifies abnormal cardiac orientation when the estimated orientation is opposite to the examiner’s assessment because it determines fetal presentation simultaneously with cardiac axis measurement.

An abnormality assessment method was further introduced based on the relative positions of the cardiac apex and the descending aorta. In normal anatomy, the apex and descending aorta are located on the same side of the thoracic cavity in both cephalic and breech positions [32]. Using this relationship, conditions in which these structures lie on opposite sides of the thoracic cavity, such as right-sided aortic arch (RAA), can be detected. When this condition is present, visceral malposition or deviation of the cardiac axis is suspected; however, this approach cannot distinguish between right- and left-sided isomerism. As shown in Figure 3, point A was defined as the intersection between the line passing through point S and the centroid of the descending aorta and the thoracic circumference on the side opposite to point S relative to the descending aorta. In the normal case, point V lies on the arc AR, which the following angular relationship can express:

(5) ∠VSA < ∠RSV+∠RSA.

**Figure 4:**
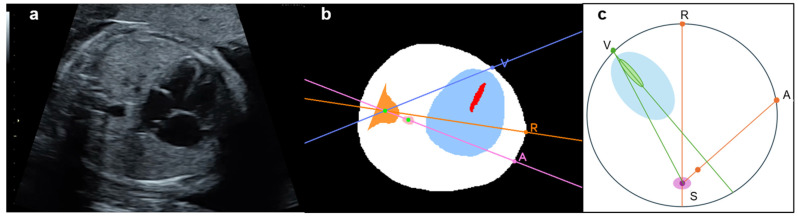
Location of the heart and descending aorta in patient with congenital heart disease (CHD) (right aortic arch). Original (**a**) and segmented and processed images (**b**). Pattern diagram indicates that the heart and descending aorta are located in the opposite thoracic cavity (**c**). A straight line drawn from point S to the other three points, respectively, is described as follows: patient with CHD: ∠VSA = ∠RSV + ∠RSA. A, The intersection of the extension pink line connecting the spine and descending aorta with the thoracic circumference as the descending aorta substitution; R, Intersection of the thoracic circumference with an orange line passing through the spine and bisecting the whole thorax; S, Spine; V, Intersection of a line through the centroid of the ventricular septum and the thoracic circumference, closer to the ventricular septum as the apex substitute. The extension purple line passes through S and V.

If the heart and descending aorta are located in opposite thoracic cavities, as shown in Figure 4, the relationship becomes:

(6) ∠VSA = ∠RSV+∠RSA.

Depending on image quality, the descending aorta may appear very close to line RS even in normal cases. However, because the primary purpose of this study was screening, no numerical threshold was applied. Abnormalities were therefore determined using the relationships defined above.

#### 2.5.3. Point P

**Figure 5:**
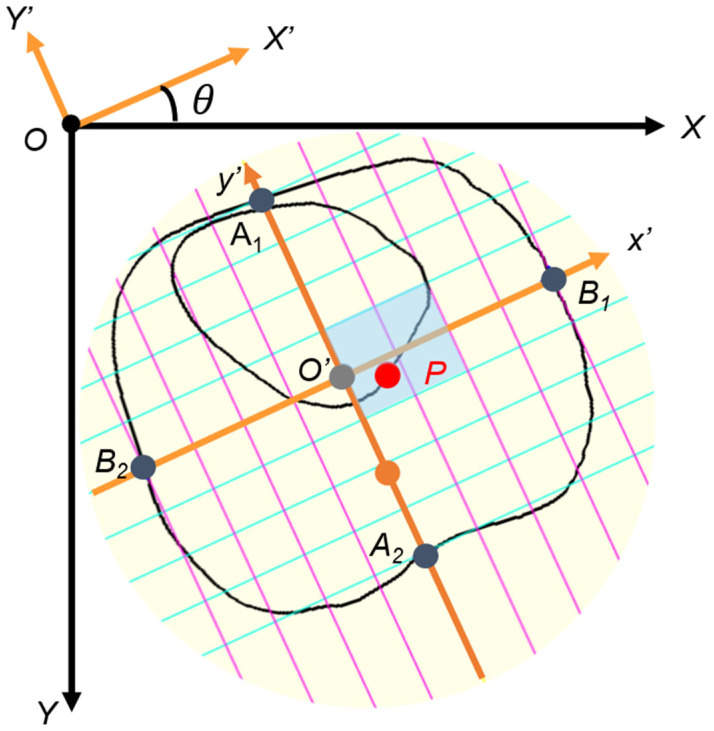
Cardiac position (point P). To construct relative coordinates, point O’ (*c_x_*, *c_y_*) is moved to origin O (0, 0) on the relative coordinate *x*- and *y*-axes. Point P (*p_x_*, *p_y_*) is similarly moved. The *x*′- and *y*′-axis are tilted by an angle *θ* due to the tilt of the thorax, so they are rotated horizontally. The *y*-axis is reversed to the negative direction. Lines *B*_1_*B*_2_ and *A*_1_*A*_2_ of the relative coordinate axes are divided into eight equal parts, and the scale is then adjusted. The gray-colored box is the normal range of point P.

Point P was originally defined as the intersection of the atrial septum and the atrial wall [36]. To approximate its location in the present study, point P (*p_x_*, *p_y_*) was defined as the intersection between line IV and the segmented cardiac circumference on the side opposite the ventricular septum. The centroid of the whole thorax was defined as point O′ (*c_x_*, *c_y_*), and the *x*′ and *y*′ axes were centered at point O′. The probability that the coordinate of point P was within (0 ≤ *p_x_* ≤ 2, −1 ≤ *p_y_* ≤ 1) was approximately 96.3% (Figure 5) [36]. In the relative coordinate axes *x*′ and *y*′, the points of intersection with the circumference of the entire thorax are defined as *B*_1_, *B*_2_, *A*_1_, and *A*_2_, respectively.

① To construct the relative coordinates, point O′ (*c_x_*, *c_y_*) is moved to the origin O (0, 0) on the relative coordinate axes *X* and *Y*. Point P (*p_x_*, *p_y_*) is moved in a similar manner.
② The *x*′ and *y*′ axes are tilted by an angle *θ* due to the tilt of the thorax, so they are rotated horizontally.
③ The *y*′ axis is reversed to the negative direction.
④ Lines *B*_1_*B*_2_ and *A*_1_*A*_2_ of the relative coordinate axes are divided into eight equal parts, and the scale is then adjusted.

(7) $$dx=\frac{1}{2}B_{1}B_{2},\;dy=\frac{1}{2}A_{1}A_{2}\;\left[\begin{array}{c} p_{x}^{\prime }\\ p_{y}^{\prime }\\ 1 \end{array}\right]=\left[\begin{array}{ccc} 1/dx & 0 & 0\\ 0 & 1/dy & 0\\ 0 & 0 & 1 \end{array}\right]\left[\begin{array}{ccc} 1 & 0 & 0\\ 0 & -1 & 0\\ 0 & 0 & 1 \end{array}\right]\left[\begin{array}{ccc} \cos\theta & -\sin\theta & 0\\ \sin\theta & \cos\theta & 0\\ 0 & 0 & 1 \end{array}\right]\left[\begin{array}{ccc} 1 & 0 & -c_{x}\\ 0 & 1 & -c_{y}\\ 0 & 0 & 1 \end{array}\right]\left[\begin{array}{c} p_{x}\\ p_{y}\\ 1 \end{array}\right]\;g_{x}=\left\{\begin{array}{c} \left\lceil p_{x}^{\prime }\right\rceil \;\;\;\;\;\;\;\;\;\;\left(p_{x}^{\prime }>0\right)\\ 0\;\;\;\;\;\;\;\;\;\;\;\;\;\left(p_{x}^{\prime }=0\right)\\ \left\lfloor p_{x}^{\prime }\right\rfloor \;\;\;\;\;\;\;\;\;\;\left(p_{x}^{\prime }<0\right) \end{array}\right.\;\;\;{\;g}_{y}=\left\{\begin{array}{c} \left\lceil p_{y}^{\prime }\right\rceil \;\;\;\;\;\;\;\;\;\;\left(p_{y}^{\prime }>0\right)\\ 0\;\;\;\;\;\;\;\;\;\;\;\;\;\left(p_{y}^{\prime }=0\right)\\ \left\lfloor p_{y}^{\prime }\right\rfloor \;\;\;\;\;\;\;\;\;\;\left(p_{y}^{\prime }<0\right) \end{array}\right.$$

The normal reference ranges were defined according to the Practice Guidelines of the International Society of Ultrasound in Obstetrics and Gynecology: CTAR < 35%, cardiac axis 45 ± 20° toward the left thorax, and point P evaluated using the relative coordinate system centered at the thoracic centroid, as described above [32,36,37]. We assessed whether point P lay outside the normal coordinate range. For the primary endpoint, parameter values obtained from the AI models were compared with those measured by obstetricians to determine equivalence.

### 2.6. Performance Evaluation of the AI Models

For automatic image extraction using YOLOv7, images were considered unsuitable for evaluation if the 4CV could not be recognized and extracted. First, image extraction was performed on 35 normal fetal cardiac ultrasound videos from 33 patients acquired using Voluson^®^ E8 or E10. Second, 10 normal fetal cardiac ultrasound videos from 10 patients acquired using the newer Voluson^®^ Expert 22 were analyzed to evaluate model generalizability across devices. Each extracted image was segmented using UNet 3+ and SegFormer, and the mDice and other parameters were calculated from the segmentation results. Because only normal cases were used to train the AI models, both the internal and external validation datasets consisted exclusively of normal cases.

To evaluate model performance in patients with CHD, 22 fetal cardiac ultrasound videos from 22 patients acquired using Voluson^®^ E8, E10, or Expert 22 were analyzed. The diagnoses included tetralogy of Fallot (TOF), Ebstein’s anomaly, hypoplastic left heart syndrome, pulmonary atresia with an intact ventricular septum, tricuspid atresia, transposition of the great arteries, congenital cystic adenomatoid malformation, congenital diaphragmatic hernia, double-outlet right ventricle, ventricular septal defect, and RAA. As in the normal cohort, YOLOv7 automatically and randomly extracted two 4CV images from each video. Videos from which no 4CV image could be extracted were classified as abnormal. The extracted images were segmented using UNet 3+ and SegFormer, and all parameters were subsequently calculated.

### 2.7. Clinical Comparison Study

A comparative study between the AI models and 22 obstetricians was conducted at Showa Medical University Hospital and Showa Medical University Koto Toyosu Hospital to determine whether parameter errors were clinically acceptable. The obstetricians were divided into three groups: experts (n = 3; 19, 24, and 31 years of experience), fellows (n = 7; 6–11 years of experience), and residents (n = 12; 3–5 years of experience). All participants were provided with fetal ultrasound videos acquired using Voluson^®^ E8, E10, or Expert 22, including 10 normal cases and 10 cases with CHD. Each obstetrician was asked to extract one thoracic image from each video that contained the optimal 4CV for measurement. To replicate the clinical workflow, each extracted image was segmented for the whole thorax and heart using the elliptical method, with a straight line bisecting the thoracic area through the spine and another bisecting the ventricular septum, and point P was marked [37,38]. The calculation algorithm then automatically derived the CTAR and cardiac axis. Point P was visually assessed for whether it lay within the normal range using the automatically generated thorax-based coordinate system. After collecting all responses, each parameter was calculated within the program.

The AI results for UNet 3+ and SegFormer were computed using 2 images extracted from the same 20 videos evaluated by the obstetricians. To assess screening performance, the results of these two models were compared with those obtained by the obstetricians. Model performance was summarized using ROC curves. After confirming homogeneity of variances using Levene’s test, one-way analysis of variance (ANOVA) or Welch’s ANOVA was applied to test for significant differences between the obstetricians’ and AI models’ parameter values (*p* < 0.05). When significant differences were detected, Tukey’s honestly significant difference test for homoscedasticity or the Games–Howell test was performed.

## 3. Results

### 3.1. Model Structure and Internal Validation

The testing dataset comprised 58 images automatically extracted from normal fetal cardiac ultrasound videos using YOLOv7. Images that could not be segmented automatically because of poor image quality were excluded from the mDice calculation for each structure. The mDice values for the heart, ventricular septum, whole thorax, thorax, and descending aorta were 0.923, 0.783, 0.949, 0.946, and 0.658, respectively, for UNet 3+ and 0.928, 0.776, 0.951, 0.949, and 0.690, respectively, for SegFormer (Table 1). For UNet 3+, the mean predictions ± standard deviation (SD) were 26.9 ± 3.5% for CTAR and 41.7 ± 9.8° for the cardiac axis, with mean absolute errors (MAEs) of 2.7% and 5.4°, respectively, relative to the ground truth. For SegFormer, the corresponding values were 27.1 ± 3.5% for CTAR and 41.6 ± 10.0° for the cardiac axis, with MAEs of 2.7% and 5.6°, respectively (Table 2). Supplementary Figure S1 shows the distribution of point P, with 90.0–95.6% of measurements falling within the normal range in the testing dataset. The performance of UNet 3+ and SegFormer was comparable.

### 3.2. Evaluation of Differences Between Ultrasound Equipment Using External Validation Dataset

For external validation to assess differences between ultrasound equipment, we analyzed 18 images automatically extracted from normal fetal cardiac ultrasound videos acquired using a different ultrasound machine with YOLOv7. As shown in Table 3, the mDice values for the heart, ventricular septum, whole thorax, thorax, and descending aorta were 0.922, 0.740, 0.941, 0.950, and 0.758, respectively, for UNet 3+ and 0.931, 0.721, 0.945, 0.944, and 0.795, respectively, for SegFormer. For biometric parameter estimation in the external validation dataset, UNet 3+ yielded values of 26.5 ± 3.9% for CTAR and 37.7 ± 14.3° for the cardiac axis, with MAEs of 2.2% and 5.0°, respectively, relative to the ground truth. SegFormer yielded values of 27.1 ± 3.8% for CTAR and 38.8 ± 13.6° for the cardiac axis, with MAEs of 2.7% and 5.0°, respectively (Table 4).

### 3.3. Evaluation Results of Images of Patients with CHD

**Figure 6:**
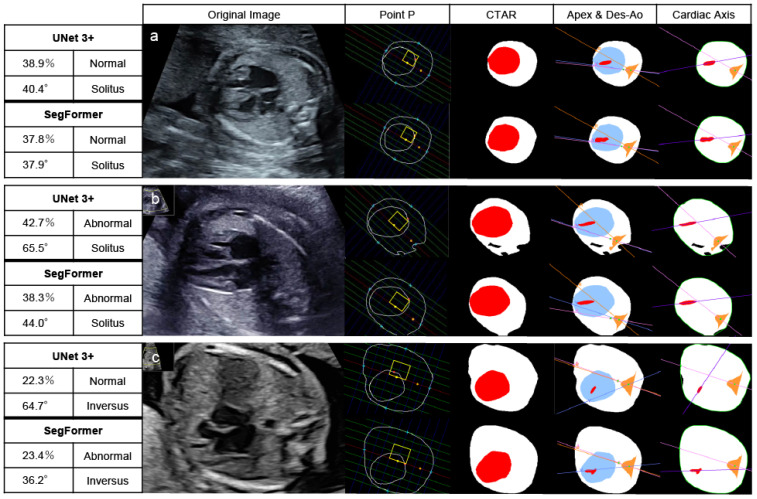
Automated assessment of the 4CV in patients with CHD. After recognizing and extracting 4CV using YOLOv7, UNet 3+ and SegFormer performed segmentation, drawing lines, and calculating parameters. Pulmonary atresia with an intact ventricular septum (23 weeks) (**a**), transposition of the great arteries (27 weeks) (**b**), and Tetralogy of Fallot (20 weeks) (**c**). These patients had at least one parameter indicating abnormalities. The yellow boxes highlight the normal range of point P. The red area represents the heart, and the white area represents the whole thorax in CTAR. Define the colored lines, the point A, R, and V in the positions of the apex and Des-Ao and the cardiac axis as shown in Figure 2 and Figure 3. In the left table of each AI model, the top left column indicates the CTAR, the top right one point P, the bottom left one the cardiac axis, and the bottom right one the positions of the apex and Des-Ao. CTAR, cardiothoracic area ratio; Des-Ao, descending aorta.

In patients with CHD, 30 images were extracted from 15 patients because 7 of the 22 patients were not recognized as having a normal 4CV and therefore could not be extracted. Of these 30 images, UNet 3+ classified 19 images and SegFormer classified 20 images as abnormal based on abnormalities in at least one of three parameters: CTAR, cardiac axis, or point P. Patients without detected abnormalities showed morphological abnormalities confined to the heart or the vascular system. Across all 22 CHD cases, the per-patient sensitivity, counting non-extractable 4CV images as screening positive, was 0.773 (17/22) for UNet 3+ and 0.818 (18/22) for SegFormer. Figure 6 shows the results of automated 4CV assessment in three patients with CHD: pulmonary atresia with an intact ventricular septum (a), transposition of the great arteries (b), and TOF (c). RAA complicates approximately 40% of patients with TOF. In this study, patients with RAA in whom the descending aorta and cardiac apex were located in opposite thoracic regions (referred to here as “inversus”) were classified as abnormal [38,39].

### 3.4. Clinical Comparison Study Between Obstetricians and AI Models

**Figure 7:**
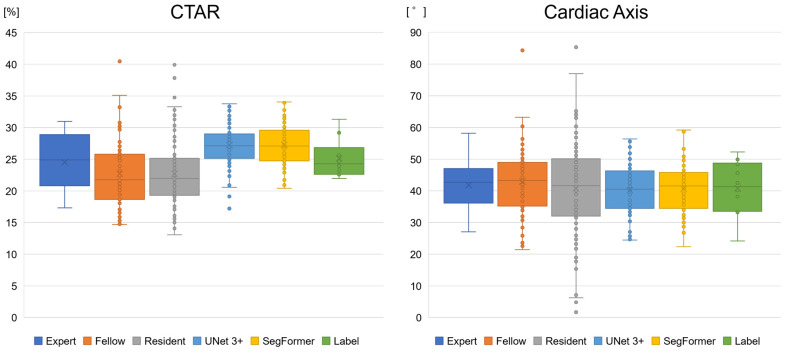
Boxplots of the biometric parameter estimation of the AI models and obstetricians. The CTAR tended to be smaller for obstetricians and larger for the AI models than for the label. However, no significant differences were observed in normal cases between the label and other groups. For the cardiac axis, no specific differences were observed. CTAR, cardiothoracic area ratio.

We compared biometric parameters derived by obstetricians and the AI models with the ground-truth labels. Supplementary Figure S2 illustrates variation in 4CV image extraction by obstetricians and YOLOv7. Experts and YOLOv7 tended to extract similar images, whereas residents showed greater variability in image selection. In 10 normal cases, the mean ± SD values for experts, fellows, and residents were 24.5 ± 4.1%, 22.5 ± 5.2%, and 22.7 ± 5.0%, respectively, for the CTAR and 41.8 ± 7.6°, 42.9 ± 11.0°, and 40.8 ± 14.7°, respectively, for the cardiac axis (Figure 7 and Table 5). For UNet 3+ and SegFormer, CTAR was 27.2 ± 3.4% and 27.3 ± 3.2%, respectively, and the cardiac axis was 40.2 ± 7.6° and 40.6 ± 7.3°, respectively. For point P, the proportion of incorrect cases in the 10 normal cases is shown in Table 5, with a maximum value of 11.7%. Compared with the labels, CTAR tended to be smaller for obstetricians and larger for the AI models. However, the Games–Howell test showed no significant differences between the label and any group in the normal cases. No significant differences were observed for the cardiac axis (Welch’s ANOVA, *p* = 0.617) (Supplementary Table S2).

### 3.5. AI Models Achieve a Screening Performance Equivalent to That of Experts

**Figure 8:**
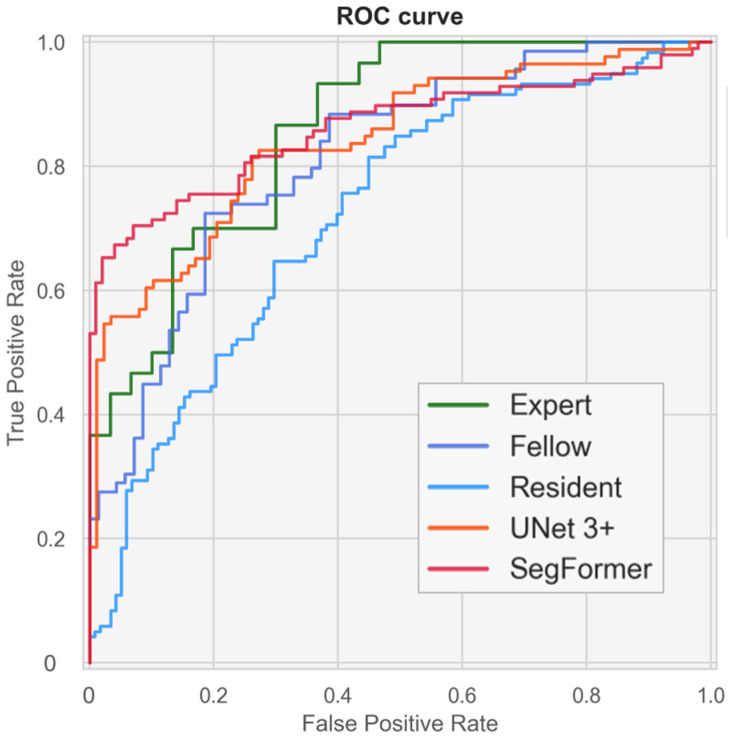
Performance of fetal cardiac ultrasound screening based on the cardiothoracic area ratio (CTAR), cardiac axis, and point P. The ROC curves illustrate the screening performances of experts, fellows, residents, and our automated models. ROC, receiver operating characteristics.

Figure 8 and Supplementary Figure S3 present ROC curves illustrating the screening performance of experts, fellows, residents, and the AI models. The area under the curve (AUC) analysis indicated that the CTAR and cardiac axis contributed more to screening performance than point P. Screening based on CTAR and the cardiac axis achieved AUC values of 0.816 (95% CI, 0.699–0.913) for experts, 0.835 (95% CI, 0.775–0.893) for UNet 3+, and 0.851 (95% CI, 0.789–0.904) for SegFormer. When the CTAR, cardiac axis, and point P were combined, the performance further improved to AUC values of 0.860 (95% CI, 0.754–0.943), 0.841 (95% CI, 0.778–0.897), and 0.861 (95% CI, 0.804–0.910) for experts, UNet 3+, and SegFormer, respectively. These results indicate that the AI models achieved performance comparable to that of experts. SegFormer achieved the same AUC as the experts, and its ROC curve intersected that of the experts (Figure 8 and Table 6). Using the Youden index for experts, the clinically acceptable false-positive rate was 0.300. At this operating point, sensitivity was 0.867 for experts and 0.816 for SegFormer. These findings suggest that experts achieved higher sensitivity, whereas SegFormer achieved higher specificity.

## 4. Discussion

The skill level of examiners does not improve rapidly, and continuous training is required for fetal cardiac ultrasound examinations [26,39]. This study was designed to simulate a clinical scenario in which an examiner can perform basic prenatal screening using an AI model. Several studies have reported that CNN models can classify individual cross-sectional frames extracted from cardiac sweep videos and detect abnormalities [27,40,41]. However, in those studies, diagnoses were not made by physicians, and even when abnormalities were detected, their underlying causes were not clearly defined. In contrast, Liang et al. applied the CTAR and cardiac axis using segmentation methods [42], and Taksøe-Vester et al. developed a segmentation-based AI model for screening fetal coarctation of the aorta [43]. In addition, AI-based automated assessment of the pulmonary artery–to–ascending aorta ratio in the 3VV has been reported [33]. Furthermore, a novel approach using three-dimensional segmentation models has been proposed for prenatal ultrasound screening of total anomalous pulmonary venous connection [44].

In this study, biometric parameter calculations were designed to align as closely as possible with the CTAR and cardiac axis measurement methods used in clinical practice, thereby facilitating acceptance of the AI system by potential users. To address the domain-shift problem, variability in ultrasound devices and gestational age was restricted. Accordingly, this study verified compatibility between a newer ultrasound device and a widely used model from the same vendor. In the automated 4CV assessment model, the screening workflow comprised the following steps: (1) automatic detection and extraction of the 4CV using YOLOv7; (2) calculation of biometric parameters, including the CTAR, cardiac axis, and point P; (3) confirmation of fetal position (cephalic or breech); and (4) confirmation of the positions of the descending aorta and cardiac apex (solitus or inversus). Examiners can screen for 4CV morphological abnormalities during the initial YOLOv7-based detection and through subsequent identification of abnormal parameter values. Screening results can be shared efficiently between examiners and experts, which may accelerate the initial evaluation and facilitate timely referral for secondary examination. Final determination of disease-relevant abnormalities, however, remains the responsibility of a skilled examiner.

We compared UNet 3+ and SegFormer and found that both achieved comparable performance in terms of mDice and parameter estimation accuracy. With respect to network architecture and computational efficiency, UNet 3+ has fewer parameters and faster inference speed, whereas SegFormer requires lower computational complexity. Both architectures were selected because they enable accurate extraction of spatial relationships during feature learning; however, segmentation performance alone did not allow a definitive determination of superiority. Nevertheless, a clinical comparison study suggested that SegFormer performed slightly better than UNet 3+. For both the CTAR and cardiac axis, the MAEs of the AI models were similar to those of obstetricians, and these discrepancies were considered clinically acceptable. Regarding the range of these parameters in normal cases, no significant differences were observed among the labels, experts, or AI models. In addition, the AI models showed accuracy comparable to that of a related study (CTAR; 31.0 ± 4.0%, cardiac axis; 32.0 ± 8.2°) [42]. Point P is rarely used in routine clinical practice because of the difficulty of manual measurement; however, the availability of computational resources and AI assistance may enable more frequent use of this parameter in the future. A clinical comparison study indicated that fetal cardiac ultrasound screening based on the CTAR, cardiac axis, and point P achieved a performance comparable to that of experts. In addition, this study demonstrated greater variability in 4CV extraction among residents than among experts, suggesting that AI-assisted biometric parameter calculation and standardized 4CV extraction may particularly benefit less experienced examiners. Our fully automated AI models can reduce missed abnormalities and standardize screening accuracy, thereby leading to improved prenatal diagnostic rates.

Although increasing the number of training images can improve model accuracy, identification of the ventricular septum and descending aorta remains challenging in low-quality images, which limits overall performance. Moreover, the fundamental method of fetal ultrasound examination is unlikely to change without major technological advances in ultrasound equipment. Automated 4CV assessment systems, including the AI models proposed here, therefore remain highly dependent on examiner performance. Images that AI easily analyzes are also generally easy for humans to interpret. Consequently, continued efforts to improve examiner skill in acquiring high-quality images remain essential. Higher quality training data will lead to further improvements in AI performance.

This study has some limitations. First, although the accuracy of the device used in this study and its newer models was verified, measurement accuracy may decline when ultrasound devices from other vendors or devices with lower image quality are used. Second, because the training data consisted of fetal cardiac ultrasound videos acquired between 18 and 28 weeks of gestation, the generalizability of the models to other gestational ages requires further validation. Fetal cardiac structures are smaller and less clear in the first trimester, making analysis more difficult to perform. However, the structures become clearer in the third trimester and the screening performance is close to that of a diagnosis. In future studies, to expand the scope of evaluation to the first and third trimesters for clinical applications, new AI models or multimodal methods should be explored. Third, interobserver and intraobserver variability in the segmentation labels was not assessed, which may have introduced bias into both the mDice and the MAE. Fourth, because images for the CTAR were randomly extracted from videos, systolic and diastolic frames were analyzed together. Fifth, the validation and test results were based on the average of two extracted images; however, the optimal number of images required for this approach remains to be determined. With continued advances in AI, automated systems are expected to extract appropriate cross-sectional views from multiple sites with fewer probe movements and to compute multiple parameters automatically. Finally, the fully automated AI models were built on existing standard architectures. Future work should explore architectural modifications that avoid potential patent infringement during clinical deployment. In addition, ablation studies should be performed when updating the architecture, using state-of-the-art YOLO-series or segmentation models.

## 5. Conclusions

We developed fully automated AI models that extract 4CV images from fetal cardiac ultrasound videos and quantify the CTAR, cardiac axis, and point P. In the clinical comparison study, screening performance based on all three biometric parameters achieved AUC values of 0.860, 0.841, and 0.861 for experts, UNet 3+, and SegFormer, respectively. These models are expected to reduce missed abnormalities and to improve the standardization of examination accuracy. In a clinical scenario, the models could support fetal cardiac ultrasound screening, with the final diagnosis made by an obstetrician when abnormal parameter values are identified. The integration of AI is also expected to enable more accurate assessments through the use of higher-quality images and to encourage examiners to acquire optimal input images. Consequently, the skill levels of examiners using these models may become both improved and more consistent.

## Figures and Tables

**Table 1:** Segmentation results for the AI models in the 4CV.

mDice	UNet 3+	SegFormer
Heart	0.923	0.928
Ventricular Septum	0.783	0.776
Whole Thorax	0.949	0.951
Thorax	0.946	0.949
Descending Aorta	0.658	0.690

mDice, the mean values of the Dice coefficient.

**Table 2:** Biometric parameter estimation based on cardiovascular segmentation.

	CTAR		Cardiac Axis	
	Mean ± SD	MAE	Mean ± SD	MAE
UNet 3+	26.9 ± 3.5	2.7	41.7 ± 9.8	5.4
SegFormer	27.1 ± 3.5	2.7	41.6 ± 10.0	5.6
Ground Truth	24.7 ± 3.7		41.4 ± 9.9	

CTAR, cardiothoracic area ratio; SD, standard deviation; MAE, mean absolute error.

**Table 3:** Segmentation results in external validation with another ultrasound machine.

mDice	UNet 3+	SegFormer
Heart	0.922	0.931
Ventricular Septum	0.740	0.721
Whole Thorax	0.941	0.945
Thorax	0.950	0.944
Descending Aorta	0.758	0.795

mDice, the mean values of the Dice coefficient.

**Table 4:** Biometric parameter estimation in external validation with another ultrasound machine.

	CTAR		Cardiac Axis	
	Mean ± SD	MAE	Mean ± SD	MAE
UNet 3+	26.5 ± 3.9	2.2	37.7 ± 14.3	5.0
SegFormer	27.1 ± 3.8	2.7	38.8 ± 13.6	5.0
Ground truth	24.7 ± 3.8		38.1 ± 13.6	

CTAR, cardiothoracic area ratio; SD, standard deviation; MAE, mean absolute error.

**Table 5:** Performance evaluation of the AI models and obstetricians for 10 normal cases in the clinical comparison study.

	CTAR	Cardiac Axis	Point P
Expert	24.5 ± 4.1	41.8 ± 7.6	1.7
Fellow	22.5 ± 5.2	42.9 ± 11.0	7.9
Resident	22.7 ± 5.0	40.8 ± 14.7	11.7
UNet 3+	27.2 ± 3.4	40.2 ± 7.6	5.0
SegFormer	27.3 ± 3.2	40.6 ± 7.3	5.0
Ground truth (label)	25.1 ± 2.9	40.8 ± 8.3	

All data are shown as mean ± SD in CTAR and cardiac axis, and mean ratio of incorrect in point P. CTAR, cardiothoracic area ratio; SD, standard deviation.

**Table 6:** Performance of fetal cardiac ultrasound screening with or without each biometric parameter in the clinical comparison study.

CTAR	Cardiac Axis	Point P	Resident	Fellow	Expert	UNet 3+	SegFormer
✓			0.538 [0.462, 0.613]	0.563 [0.467, 0.659]	0.597 [0.442, 0.740]	0.712 [0.625, 0.790]	0.712 [0.632, 0.780]
	✓		0.702 [0.632, 0.763]	0.743 [0.657, 0.818]	0.783 [0.656, 0.890]	0.743 [0.653, 0.816]	0.778 [0.711, 0.838]
✓	✓		0.705 [0.637, 0.764]	0.748 [0.656, 0.824]	0.816 [0.699, 0.913]	0.835 [0.775, 0.893]	0.851 [0.789, 0.904]
✓	✓	✓	0.722 [0.657, 0.779]	0.814 [0.738, 0.880]	0.860 [0.754, 0.943]	0.841 [0.778, 0.897]	0.861 [0.804, 0.910]

All data are shown as mean AUC [95% CI]. Check marks indicate the meaning used for screening. AUC, area under the curve; CTAR, cardiothoracic area ratio.

## Data Availability

The datasets generated and/or analyzed during the current study are not publicly available to preserve the privacy of the participants.

## Supplementary Materials

The following supporting information can be downloaded at: https://www.mdpi.com/article/10.3390/bioengineering13030303/s1, Figure S1: Distribution of all plots of points P in the testing dataset; Figure S2: Variation of the 4CV image extraction; Figure S3: ROC curves of fetal cardiac ultrasound screening with or without each biometric parameter in the clinical comparison study; Table S1: Details of the dataset; Table S2: Statistical differences in parameter values between different groups of obstetricians and AI models.

## Abbreviations

The following abbreviations are used in this manuscript:

AI	Artificial intelligence
ANOVA	Analysis of variance
AUC	Area under the curve
CHD	Congenital heart disease
CNN	Convolutional neural network
CTAR	Cardiothoracic area ratio
FN	False negatives
FP	False positives
IoU	Intersection over union
MAE	Mean absolute errors
mDice	Mean values of the dice coefficient
MS-SSIM	Multiscale structural similarity index
Point P	Cardiac position
ROC	Receiver operating characteristic
SD	Standard deviation
TOF	Tetralogy of Fallot
TP	True positives
YOLO	You only look once
3VV 4CV	Three-vessel view Four-chamber view

## References

[1] Lee L.H., Bradburn E., Craik R., Yaqub M., Norris S.A., Ismail L.C., Ohuma E.O., Barros F.C., Lambert A., Carvalho M. (2023). Machine learning for accurate estimation of fetal gestational age based on ultrasound images. npj Digit. Med..

[2] He F., Li G., Zhang Z., Yang C., Yang Z., Ding H., Zhao D., Sun W., Wang Y., Zeng K. (2025). Transfer learning method for prenatal ultrasound diagnosis of biliary atresia. npj Digit. Med..

[3] Meng Q., Sinclair M., Zimmer V., Hou B., Rajchl M., Toussaint N., Oktay O., Schlemper J., Gomez A., Housden J. (2019). Weakly Supervised Estimation of Shadow Confidence Maps in Fetal Ultrasound Imaging. IEEE Trans. Med. Imaging.

[4] Campanella G., Hanna M.G., Geneslaw L., Miraflor A., Werneck Krauss Silva V., Busam K.J., Brogi E., Reuter V.E., Klimstra D.S., Fuchs T.J. (2019). Clinical-grade computational pathology using weakly supervised deep learning on whole slide images. Nat. Med..

[5] Yamada Y., Kobayashi M., Shinkawa K., Bilal E., Liao J., Nemoto M., Ota M., Nemoto K., Arai T. (2025). Utility of synthetic musculoskeletal gaits for generalizable healthcare applications. Nat. Commun..

[6] Rao V.M., Hla M., Moor M., Adithan S., Kwak S., Topol E.J., Rajpurkar P. (2025). Multimodal generative AI for medical image interpretation. Nature.

[7] You J., Zhang S., Zhang J., Chen Y., Zhang M., Zhou C., Jiang B. (2025). Ensemble learning for predicting microsatellite instability in colorectal cancer using pretreatment colonoscopy images and clinical data. Front. Oncol..

[8] Khullar V., Abbas M., Kansal I., Ksibi A., Gupta G., Gupta D., Juneja S., Nauman A. (2026). Low resource federated learning for classification of nail disease by deploying cross-silo and heterogeneously dataset distributions. Sci. Rep..

[9] Wang Z., Yang Y., Chen Y., Yuan T., Sermesant M., Delingette H., Wu O. (2024). Mutual Information Guided Diffusion for Zero-Shot Cross-Modality Medical Image Translation. IEEE Trans. Med. Imaging.

[10] Wang W., Li Y., Lu K., Zhang J., Chen P., Yan K., Wang B. (2024). Medical Tumor Image Classification Based on Few-Shot Learning. IEEE/ACM Trans. Comput. Biol. Bioinform..

[11] Komatsu M., Komatsu R., Sakai A., Yasutomi S., Harada N., Aoyama R., Teraya N., Takeda K., Natsume T., Taniguchi T. (2025). Establishment of High-Precision Ultrasound Diagnosis Methods Based on the Introduction of Deep Learning. IEEE Rev. Biomed. Eng..

[12] Fiorentino M.C., Villani F.P., Di Cosmo M., Frontoni E., Moccia S. (2023). A review on deep-learning algorithms for fetal ultrasound-image analysis. Med. Image Anal..

[13] Zhang J., Xiao S., Zhu Y., Zhang Z., Cao H., Xie M., Zhang L. (2024). Advances in the Application of Artificial Intelligence in Fetal Echocardiography. J. Am. Soc. Echocardiogr..

[14] Slimani S., Hounka S., Mahmoudi A., Rehah T., Laoudiyi D., Saadi H., Bouziyane A., Lamrissi A., Jalal M., Bouhya S. (2023). Fetal biometry and amniotic fluid volume assessment end-to-end automation using Deep Learning. Nat. Commun..

[15] Xie H.N., Wang N., He M., Zhang L.H., Cai H.M., Xian J.B., Lin M.F., Zheng J., Yang Y.Z. (2020). Using deep-learning algorithms to classify fetal brain ultrasound images as normal or abnormal. Ultrasound Obstet. Gynecol..

[16] Burgos-Artizzu X.P., Coronado-Gutiérrez D., Valenzuela-Alcaraz B., Bonet-Carne E., Eixarch E., Crispi F., Gratacós E. (2020). Evaluation of deep convolutional neural networks for automatic classification of common maternal fetal ultrasound planes. Sci. Rep..

[17] He B., Kwan A.C., Cho J.H., Yuan N., Pollick C., Shiota T., Ebinger J., Bello N.A., Wei J., Josan K. (2023). Blinded, randomized trial of sonographer versus AI cardiac function assessment. Nature.

[18] Frydman S., Freund O., Miller R., Sror N., Barel N., Baruch G., Rothschild E., Merin R., Shporn O., Ohad M. (2026). The effect of AI-assisted bedside echocardiography on inpatient care: A prospective trial. Eur. Heart J. Digit. Health.

[19] He X., Mohamed M.O., Ng N.Y.J., Kumaran T., Bajaj R., Yap N.A.L., Erdogan E., Zeren G., Mathur A., Ulutas A.E. (2026). A deep learning methodology for fully-automated quantification of calcific burden in high-resolution intravascular ultrasound images. Int. J. Cardiovasc. Imaging.

[20] Wu W., He J., Shao X. (2020). Incidence and mortality trend of congenital heart disease at the global, regional, and national level, 1990–2017. Medicine.

[21] Bouma B.J., Mulder B.J. (2017). Changing Landscape of Congenital Heart Disease. Circ. Res..

[22] Matsui H., Hirata Y., Inuzuka R., Hayashi T., Nagamine H., Ueda T., Nakayama T. (2021). Initial national investigation of the prenatal diagnosis of congenital heart malformations in Japan-Regional Detection Rate and Emergency Transfer from 2013 to 2017. J. Cardiol..

[23] Aldridge N., Pandya P., Rankin J., Miller N., Broughan J., Permalloo N., McHugh A., Stevens S. (2023). Detection rates of a national fetal anomaly screening programme: A national cohort study. Bjog Int. J. Obstet. Gynaecol..

[24] Oggè G., Gaglioti P., Maccanti S., Faggiano F., Todros T. (2006). Prenatal screening for congenital heart disease with four-chamber and outflow-tract views: A multicenter study. Ultrasound Obstet. Gynecol..

[25] van Nisselrooij A.E.L., Teunissen A.K.K., Clur S.A., Rozendaal L., Pajkrt E., Linskens I.H., Rammeloo L., van Lith J.M.M., Blom N.A., Haak M.C. (2020). Why are congenital heart defects being missed?. Ultrasound Obstet. Gynecol..

[26] Diniz P.H.B., Yin Y., Collins S. (2020). Deep Learning strategies for Ultrasound in Pregnancy. Eur. Med. J. Reprod. Health.

[27] Arnaout R., Curran L., Zhao Y., Levine J.C., Chinn E., Moon-Grady A.J. (2021). An ensemble of neural networks provides expert-level prenatal detection of complex congenital heart disease. Nat. Med..

[28] Komatsu M., Sakai A., Komatsu R., Matsuoka R., Yasutomi S., Shozu K., Dozen A., Machino H., Hidaka H., Arakaki T. (2021). Detection of Cardiac Structural Abnormalities in Fetal Ultrasound Videos Using Deep Learning. Appl. Sci..

[29] Huang H., Lin L., Tong R., Hu H., Zhang Q., Iwamoto Y., Han X., Chen Y., Wu J. (2020). UNet 3+: A Full-Scale Connected UNet for Medical Image Segmentation. Proceedings of the ICASSP 2020—2020 IEEE International Conference on Acoustics, Speech and Signal Processing (ICASSP).

[30] Xie E., Wang W., Yu Z., Anandkumar A., Álvarez J.M., Luo P. SegFormer: Simple and Efficient Design for Semantic Segmentation with Transformers. Proceedings of the Neural Information Processing Systems.

[31] Wang C.-Y., Bochkovskiy A., Liao H.-Y.M. (2022). YOLOv7: Trainable Bag-of-Freebies Sets New State-of-the-Art for Real-Time Object Detectors. Proceedings of the 2023 IEEE/CVF Conference on Computer Vision and Pattern Recognition (CVPR).

[32] Carvalho J.S., Axt-Fliedner R., Chaoui R., Copel J.A., Cuneo B.F., Goff D., Gordin Kopylov L., Hecher K., Lee W., Moon-Grady A.J. (2023). ISUOG Practice Guidelines (updated): Fetal cardiac screening. Ultrasound Obstet. Gynecol..

[33] Aoyama R., Komatsu M., Harada N., Komatsu R., Sakai A., Takeda K., Teraya N., Asada K., Kaneko S., Iwamoto K. (2024). Automated Assessment of the Pulmonary Artery-to-Ascending Aorta Ratio in Fetal Cardiac Ultrasound Screening Using Artificial Intelligence. Bioengineering.

[34] Lin T.Y., Goyal P., Girshick R., He K., Dollár P. Focal Loss for Dense Object Detection. Proceedings of the 2017 IEEE International Conference on Computer Vision (ICCV).

[35] Wang Z., Simoncelli E.P., Bovik A.C. Multiscale structural similarity for image quality assessment. Proceedings of the The Thrity-Seventh Asilomar Conference on Signals, Systems & Computers 2003.

[36] Inamura N., Horigome H., Takigiku K., Shibuya K., Yoda H., Kawazu Y., Hirono K., Maeno Y., Suda K., Kawataki M. (2023). Guidelines for Fetal Echocardiography (Second Edition). J. Pediatr. Cardiol. Card. Surg..

[37] Donofrio M.T., Moon-Grady A.J., Hornberger L.K., Copel J.A., Sklansky M.S., Abuhamad A., Cuneo B.F., Huhta J.C., Jonas R.A., Krishnan A. (2014). Diagnosis and treatment of fetal cardiac disease: A scientific statement from the American Heart Association. Circulation.

[38] Awadh A.M., Prefumo F., Bland J.M., Carvalho J.S. (2006). Assessment of the intraobserver variability in the measurement of fetal cardiothoracic ratio using ellipse and diameter methods. Ultrasound Obstet. Gynecol..

[39] Reddy C.D., Van den Eynde J., Kutty S. (2022). Artificial intelligence in perinatal diagnosis and management of congenital heart disease. Semin. Perinatol..

[40] Nurmaini S., Partan R.U., Bernolian N., Sapitri A.I., Tutuko B., Rachmatullah M.N., Darmawahyuni A., Firdaus F., Mose J.C. (2022). Deep Learning for Improving the Effectiveness of Routine Prenatal Screening for Major Congenital Heart Diseases. J. Clin. Med..

[41] Athalye C., van Nisselrooij A., Rizvi S., Haak M.C., Moon-Grady A.J., Arnaout R. (2024). Deep-learning model for prenatal congenital heart disease screening generalizes to community setting and outperforms clinical detection. Ultrasound Obstet. Gynecol..

[42] Liang B., Peng F., Luo D., Zeng Q., Wen H., Zheng B., Zou Z., An L., Wen H., Wen X. (2024). Automatic segmentation of 15 critical anatomical labels and measurements of cardiac axis and cardiothoracic ratio in fetal four chambers using nnU-NetV2. BMC Med. Inform. Decis. Mak..

[43] Taksøe-Vester C.A., Mikolaj K., Petersen O.B.B., Vejlstrup N.G., Christensen A.N., Feragen A., Nielsen M., Svendsen M.B.S., Tolsgaard M.G. (2024). Role of artificial-intelligence-assisted automated cardiac biometrics in prenatal screening for coarctation of aorta. Ultrasound Obstet. Gynecol..

[44] Komatsu R., Komatsu M., Takeda K., Harada N., Teraya N., Wakisaka S., Natsume T., Taniguchi T., Aoyama R., Kaneko M. (2026). Three-Dimensional Visualization and Detection of the Pulmonary Venous-Left Atrium Connection Using Artificial Intelligence in Fetal Cardiac Ultrasound Screening. Bioengineering.

