# Supplementary material for: Artificial Intelligence-Based Automated Assessment of the Four-Chamber View in Fetal Cardiac Ultrasound Videos
Source: Bioengineering (Basel). 2026 Mar 5;13(3):303. doi: 10.3390/bioengineering13030303 (PMC13023647; doi:10.3390/bioengineering13030303)
Supplement: Supplementary file 1 [file bioengineering-13-00303-s001.zip › bioengineering-4184964-supplementary.pdf]

Supplementary information for

Artificial intelligence-based automated assessment of the four-chamber view in fetal cardiac ultrasound videos

Naoki Teraya, Masaaki Komatsu, Katsuji Takeda, Kanto Shozu, Naoaki Harada, Reina Komatsu, Akira Sakai, Rina Aoyama, Mayumi Kaneko, Ken Asada, Syuzo Kaneko, Kazuki Iwamoto, Akitoshi Nakashima, Ryu Matsuoka, Akihiko Sekizawa & Ryuji Hamamoto

The file contains
Supplementary Figures S1-S3 and Supplementary Table S1-S2

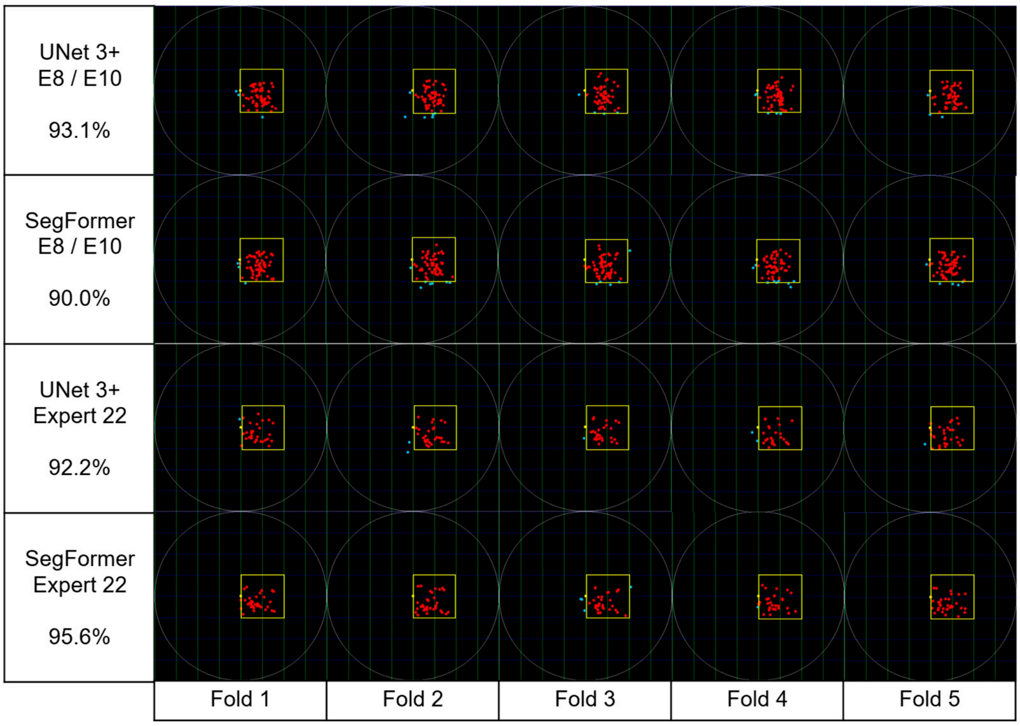

Supplementary Figure S1. Distribution of all plots of points P in the testing dataset. All segmented and evaluated images were rescaled. Moreover, 90.0–95.6% were located within the normal range. Little differences exist between UNet 3+ and SegFormer. The yellow boxes are added later to highlight the normal range of point P. The red dots are within the normal range, and the blue dots are outside it.

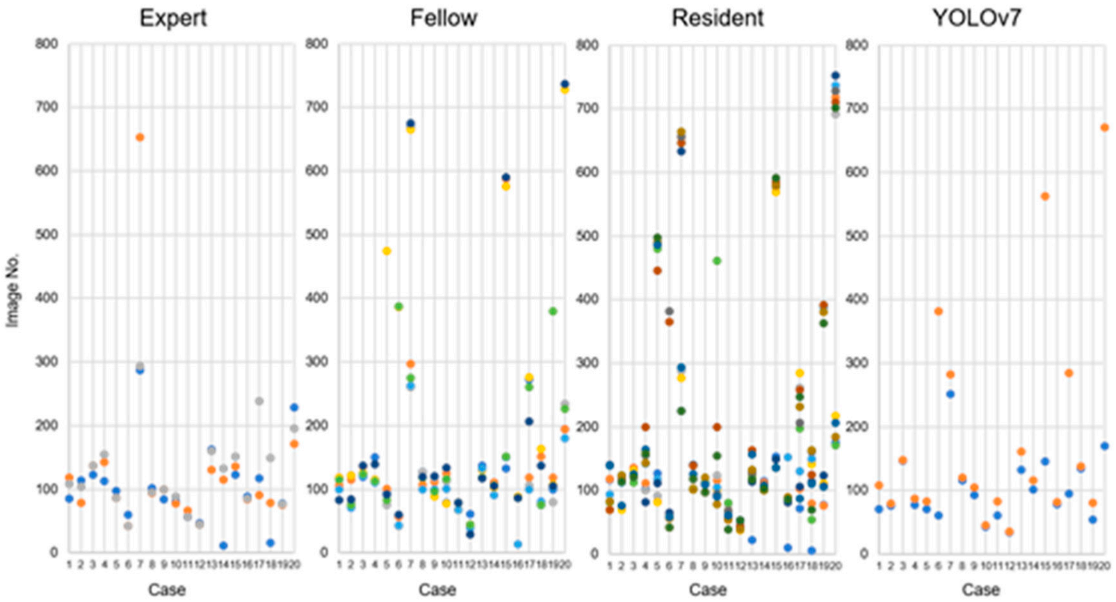

Supplementary Figure S2. Variation of the 4CV image extraction. They represent image extract numbers by the obstetricians and YOLOv7. Experts and YOLOv7 tended to extract similar images, but residents extracted various images.

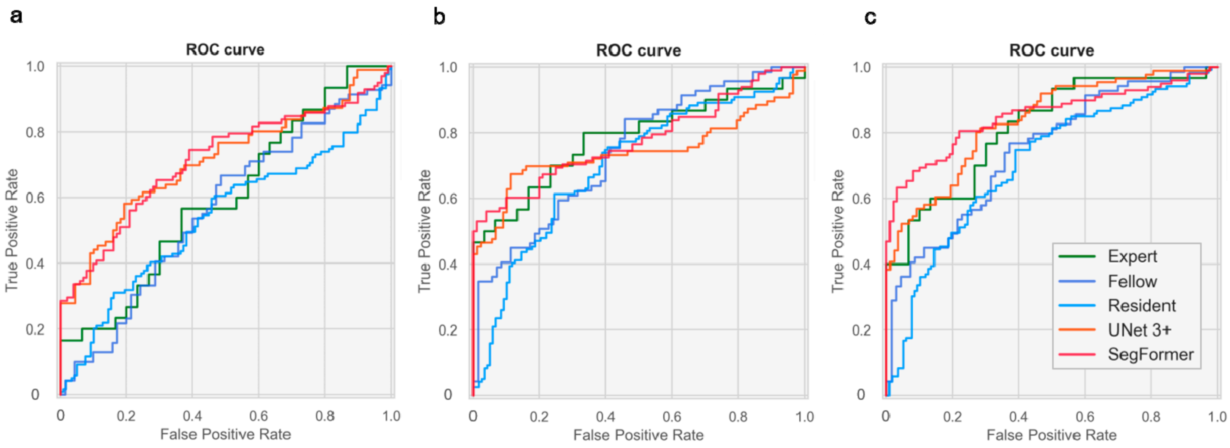

Supplementary Figure S3. ROC curves of fetal cardiac ultrasound screening with or without each biometric parameter in the clinical comparison study. ROC curves with CTAR (**a**). Those with the cardiac axis (**b**). Those with CTAR and cardiac axis (**c**). The screening performance was better using two parameters than one parameter.

Supplementary Table S1. Details of the dataset.

	**Train/Validation data**	**Test data**	**Another ultrasound machine**	**CHD**
Cases	231	33	10	22
Videos	253	35	10	22
Images	488	58	18	30
Gestational week (mean ± SD)	20.3 ± 1.7	20.3 ± 1.5	18.7 ± 0.8	22.0 ± 0

CHD, congenital heart disease; SD, standard deviation.

Supplementary Table S2. Statistical differences in parameter values between different groups of obstetricians and AI models.

[*P* values]	**CTAR**	**Cardiac Axis**
Levene's Test	< 0.0001	< 0.0001
Welch's ANOVA	< 0.0001	0.617
Games-Howell test		
SegFormer vs label	0.342	–
SegFormer vs expert	0.022	–
SegFormer vs fellow	< 0.001	–
SegFormer vs resident	< 0.001	–
SegFormer vs UNet 3+	1.000	–
UNet 3+ vs label	0.364	–
UNet 3+ vs expert	0.026	–
UNet 3+ vs fellow	< 0.001	–
UNet 3+ vs resident	< 0.001	–
expert vs label	0.996	–
expert vs fellow	0.466	–
expert vs resident	0.316	–
fellow vs label	0.343	–
fellow vs resident	1.000	–

ANOVA, analysis of variance.
